# Crystallographic Engineering of CrN Buffer Layers for GaN Thin Film Epitaxy

**DOI:** 10.3390/ma18081817

**Published:** 2025-04-16

**Authors:** Kyu-Yeon Shim, Seongho Kang, Min-Joo Ahn, Yukyeong Cha, Eojin-Gyere Ham, Dohoon Kim, Dongjin Byun

**Affiliations:** Department of Materials Science and Engineering, Korea University, 145 Anam-ro, Seongbuk-gu, Seoul 02841, Republic of Korea; ketchup@korea.ac.kr (K.-Y.S.); kangsh19427@korea.ac.kr (S.K.); ahn10170415@gmail.com (M.-J.A.); ckdbrud28@korea.ac.kr (Y.C.); rufp220@korea.ac.kr (E.-G.H.); dohoon0918@korea.ac.kr (D.K.)

**Keywords:** gallium nitride (GaN), chrome nitride (CrN), metal organic chemical vapor deposition (MOCVD), buffer layer, epitaxial growth

## Abstract

Gallium nitride (GaN) is commonly used in various semiconductor systems owing to its high mobility and thermal stability; however, the production of GaN thin films using the currently employed methods requires improvement. To facilitate the growth of high-quality GaN epitaxial thin films, this study explored the crystallographic structures, properties, and influences of chromium nitride (CrN) buffer layers sputtered under various conditions. The crystallographic orientation of CrN played a crucial role in determining the GaN film quality. For example, even when the crystallinity of the CrN (111) plane was relatively low, a single-phase CrN (111) buffer layer could provide a more favorable template for GaN epitaxy compared to cases where both the CrN (111) and Cr_2_N (110) phases coexisted. The significance of a low-temperature (LT) GaN nucleation layer deposited onto the CrN buffer layers was assessed using atomic force microscopy and contact angle measurements. The X-ray phi scan results confirmed the six-fold symmetry of the grown GaN, further emphasizing the contribution of an LT-GaN nucleation layer. These findings offer insights into the underlying mechanisms governing GaN thin film growth and provide guidance for the optimization of the buffer layer conditions to achieve high-quality GaN epitaxial films.

## 1. Introduction

Gallium nitride (GaN) is a binary III-V material with a direct band gap of 3.41 eV. Owing to its high mobility and thermal stability, GaN is widely used in various semiconductor systems, such as light-emitting diodes (LEDs), laser diodes, and high-power transistors [1,2,3]. Its growth is primarily achieved using heteroepitaxial methods. For example, the metal–organic chemical vapor deposition (MOCVD) method yields high-quality epi-layers for device fabrication. Moreover, hydride vapor-phase epitaxy results in bulk GaN substrates owing to its rapid growth rate and cost-effectiveness [4,5,6]. Frequently employed in III-nitride semiconductor fabrication, sapphire wafers (α-Al_2_O_3_) possess a hexagonal crystal structure similar to that of GaN. These materials exhibit a comparable crystallographic orientation in the (002) plane, thereby facilitating compatibility and epitaxial growth between the two compounds. However, the lattice difference between GaN and the sapphire substrates is approximately 16%, resulting in defects such as lattice mismatch and threading dislocations at the start of growth [1,7]. Therefore, employing an appropriate buffer layer, such as low-temperature (LT) GaN, aluminum nitride (AlN), or chromium nitride (CrN), is essential to reduce the lattice mismatch between the two materials and achieve high GaN crystallinity [8,9,10,11,12]. In particular, CrN is considered an effective buffer layer for GaN epitaxy, with its in-plane lattice parameter and thermal expansion coefficient lying between those of GaN and sapphire [13,14]. This structural and thermal compatibility reduces the lattice mismatch at the GaN/sapphire interface and alleviates thermal stress during the epitaxial growth process. In addition, CrN enables polarity control through straightforward surface pretreatment or the introduction of an inversion layer [15]. These combined attributes establish CrN as a technically viable and strategically advantageous buffer material for the epitaxial growth of high-quality GaN films.

Recent research has aimed to enhance the heat dissipation and light extraction efficiency of GaN-based LED devices by separating the epitaxially grown template from the sapphire substrate [16,17]. Laser and chemical lift-off (LLO and CLO, respectively) are the two primary methods employed to detach the epitaxial layers of vertical LED chips from their substrates. LLO involves irradiating the interface between the GaN layer and sapphire substrate with a laser to induce the rapid thermal decomposition of GaN, resulting in a high etching rate [18,19,20]. However, owing to the instantaneous high temperature and released nitrogen gases during decomposition, potential film deformation is a limitation of this approach [21,22,23,24]. In contrast, CLO uses a chemical solution to etch the sacrificial layer [25,26,27]. Although the substrate separation rate is lower in CLO than in LLO, CLO can selectively etch the sacrificial layer, thus avoiding damage to the epitaxial layer [27,28]. For AlN buffer layers used in LED devices, the etching rate in the chemical solution, such as potassium hydroxide, is low [28,29] and can result in the unintended etching of the GaN epitaxial layer because both materials are part of the nitride family. Thus, a stable lift-off process that does not damage the epitaxial layer is crucial. Applying CrN as both a buffer and sacrificial layer for GaN growth enables the chemical solution to selectively etch the CrN sacrificial layer without damaging the GaN epitaxial layer [12,14,30,31]. Therefore, CrN is a promising material that can simultaneously function as a buffer for GaN growth and serve as a sacrificial layer in the CLO process [32,33]. However, further research is needed to optimize the deposition of the CrN buffer layer and the corresponding conditions for GaN thin film growth.

In this study, we investigated the characteristics of sputtered CrN buffer layers under various conditions to facilitate the growth of high-quality GaN epitaxial thin films. CrN buffer layers were deposited onto sapphire (0001) substrates using reactive radio frequency (RF) magnetron sputtering under different parameters, such as the deposition temperature, nitrogen gas ratio, process pressure, and RF power. GaN was subsequently grown on the CrN buffer layers using MOCVD, and its crystallinity was compared and analyzed according to the different CrN sputtering conditions. We also investigated the impact of surface roughness on the mechanism of GaN growth by applying an LT-GaN nucleation layer during the growth process. By systematically analyzing the effects of the CrN buffer layer deposition conditions on GaN’s crystallinity, this research aimed to contribute to buffer layer design strategies for high-quality GaN epitaxy.

## 2. Materials and Methods

The CrN buffer layer deposition process was conducted using reactive RF magnetron sputtering in a high-vacuum chamber under a base pressure of 1.0 × 10^−6^ Torr. The c-plane sapphire wafer was cut to a size of 1 cm^2^; ultrasonically cleaned in acetone, isopropyl alcohol, and deionized water for 5 min each to remove impurities; and dried using N_2_ gas. CrN films (thickness = 20 nm) were deposited using a Cr (99.95% purity) target, and a mixture of Ar and N_2_ (99.9999% purity for both gases) was used as the sputtering gas. The CrN buffer layers were prepared by modulating certain parameters, including the deposition temperature, N_2_ gas ratio, process pressure, and RF power. Following CrN buffer layer deposition, GaN layers were grown using a homemade horizontal MOCVD system. The initial LT-GaN layer (grown for 1 min) was used as a nucleation layer between the CrN buffer and GaN epilayer. Trimethylgallium and ammonia (NH_3_; 99.9995% purity) were used as the Ga and N precursors, respectively, with hydrogen (H_2_; 99.9999% purity) as the carrier gas. V/III ratios of 17,252 and 4792 were applied for the LT-GaN and GaN epitaxial layers, respectively. The growth temperatures were set at 600 and 1060 °C for LT-GaN and high-temperature (HT) GaN, respectively. The effects of the CrN buffer layer deposition conditions on GaN epitaxial layers grown on sapphire substrates were investigated using X-ray diffraction (XRD; D/MAX-2500V, Rigaku, Tokyo, Japan) analysis, focusing on the crystallographic structure, growth orientation, and full-width at half maximum (FWHM) of the thin films. The surface roughness and morphology of both the previously prepared CrN layer and the CrN layer with LT-GaN growth were examined using atomic force microscopy (AFM; NX10, Park Systems, Suwon, Republic of Korea), while the surface energy was assessed using contact angle measurements (DSA-100, KRÜSS, Hamburg, Germany). The morphology of the GaN grown on buffer layers was observed using scanning electron microscopy (SEM; CX-200, COXEM, Daejeon, Republic of Korea) equipped with an energy-dispersive X-ray spectroscopy (EDX) system for compositional analysis, and the lateral alignment of GaN in the two samples was confirmed using φ-scan measurements via high-resolution XRD (Empyrean, Malvern Panalytical, Almelo, The Netherlands) analysis.

## 3. Results and Discussion

**Figure 1:**
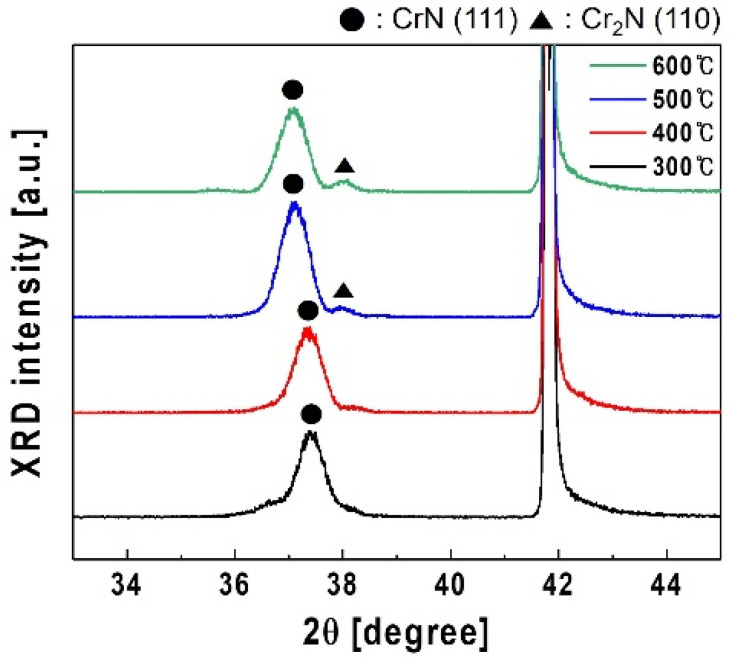
XRD patterns of CrN thin films deposited at various temperatures.

Figure 1 shows the XRD patterns of the CrN thin films on the sapphire substrates at different temperatures. The peak of the CrN thin film was dominant at approximately 37.5°, indicating that the thin film preferentially grew in the cubic-CrN (111) direction in all samples. At temperatures > 500 °C, the deposited samples exhibited a Cr_2_N (110) peak near 37.8°, suggesting a mixed phase of CrN and Cr_2_N. Notably, the Cr_2_N phase was more dominant than the cubic-CrN phase owing to the inherent characteristics of CrN thin films at high deposition temperatures [34,35].

The lattice mismatch values of the CrN thin films with sapphire and GaN (Table 1) were calculated using Bragg’s law from the (111) peak positions of the CrN samples at different temperatures, based on the XRD data shown in Figure 1. As the deposition temperature increased, the (111) peak position shifted to lower 2θ values, indicating an increase in both d-spacing and the lattice constant. Consequently, higher deposition temperatures resulted in greater lattice mismatch with the sapphire substrate but reduced mismatch with GaN. Considering these trends and the appearance of the Cr_2_N (110) peak alongside cubic-CrN (111) at temperatures of >500 °C, 400 °C was identified as a promising deposition temperature for the CrN buffer layer.

**Figure 2:**
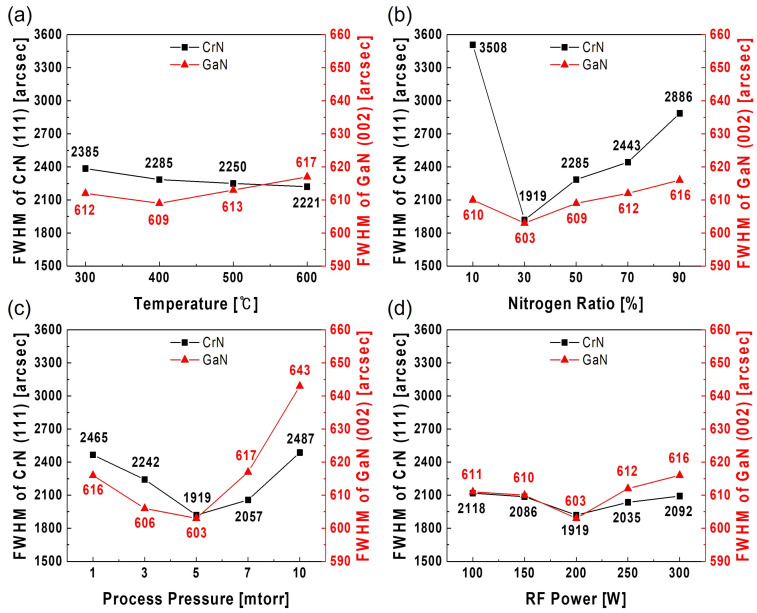
Crystallographic analyses of the CrN buffer layers and GaN thin films under varying (**a**) deposition temperatures, (**b**) nitrogen gas ratios, (**c**) process pressures, and (**d**) RF power in sputtering conditions.

GaN thin films were subsequently grown using MOCVD on CrN buffer layers sputtered at 200, 300, 400, and 500 °C. Figure 2a shows the FWHM values of CrN (111) and GaN (002) as a function of the CrN buffer layer sputtering temperature. The FWHM of CrN (111) decreased with increasing temperatures, while that of GaN (002) decreased up to 400 °C and then increased beyond 500 °C. The CrN (111) crystallinity at higher temperatures was improved; however, the CrN buffer layer formed at these temperatures theoretically reduced the lattice mismatch with GaN, suggesting the potential enhancement of GaN thin films’ crystallinity (Table 1). Despite this expectation, Figure 1 demonstrates that the CrN buffer layers that formed at >500 °C comprised a mixed phase of CrN (111) and Cr_2_N (110), rather than the CrN (111) single phase. Thus, the mixed phase degraded the crystallinity of GaN (002), resulting in GaN thin films with reduced crystallographic quality. Cr_2_N was observed in films deposited above 500 °C, consistent with reports of CrN decomposition at elevated temperatures. The Cr_2_N phase was identified based solely on XRD, with the peak at 37.834° matching the (110) reflection of the JCPDS Card (No. 79-2159). As its presence correlated with reduced GaN crystalline quality, subsequent experiments employed lower deposition temperatures to promote CrN (111) single-phase growth and suppress Cr_2_N formation. Although it is not yet clear whether a pure CrN (111), Cr_2_N (110), or mixed phase is most suitable for GaN epitaxy, our experimental results indicate that the presence of a CrN/Cr_2_N mixture leads to degraded GaN crystalline quality. This degradation may be attributed to structural disorder introduced by Cr_2_N, which has been associated with an increased grain boundary density and reduced crystallinity in Cr-based films [34]. Furthermore, Cr_2_N has been reported to exhibit irregular domain formation and poor surface planarity [36], which may contribute to non-uniform GaN nucleation and hinder epitaxial alignment. While our results suggest a correlation between Cr_2_N’s presence and degraded GaN crystallinity, the underlying mechanism remains to be fully understood and warrants further investigation.

Building upon these findings, CrN sputtering was then performed under varying nitrogen gas ratios, process pressures, and RF powers at a deposition temperature of 400 °C. To evaluate the impact of these variations, the crystallographic quality of the resulting GaN thin films was analyzed. While the CrN (111) peak positions were thoroughly analyzed under varying temperature conditions to optimize the buffer layer, subsequent experiments involving other sputtering parameters focused primarily on evaluating the resulting GaN crystallinity, and thus CrN peak shifts were not tabulated for these cases. Figure 2b shows that the CrN buffer layer exhibited a progressive reduction in the FWHM value as the nitrogen gas ratio decreased from 90% to 30%, reaching its minimum FWHM value at 30%. A further reduction to 10% resulted in an increased FWHM value, indicating a decline in crystallinity. Therefore, the CrN buffer layer exhibited the highest crystallinity under a nitrogen gas ratio of 30%. Additionally, the FWHM value of GaN (002) attained its lowest value under the 30% nitrogen gas ratio, indicating that this ratio offered the optimal conditions for CrN buffer layer deposition.

Under the optimized conditions of a 400 °C and 30% deposition temperature and nitrogen gas ratio, respectively, CrN buffer layers were deposited at various process pressures ranging from 1 to 7 mTorr in increments of 2 mTorr to evaluate their effects on the crystallinity of the CrN and GaN layers. Figure 2c shows that both the CrN (111) and GaN (002) peaks exhibited the lowest FWHM values at 5 mTorr, indicating the highest crystallinity for both layers. As the process pressure decreased, the amount of residual gas in the vacuum chamber decreased, thereby increasing the mean free path (MFP). Consequently, target ions could be deposited on the substrate with sufficient energy to enhance the crystallinity. However, at low pressures (1–3 mTorr), the scarcity of gas molecules in the vacuum chamber reduced the likelihood of electron collisions required for ionization, thus lowering the plasma density. Moreover, it decreased the number of sputtered particles that could reach the substrate, thereby reducing the deposition rate and limiting the energy available for adatom surface diffusion. Thus, the CrN buffer layer was formed with reduced atomic ordering, diminishing its crystallinity. Conversely, at high pressures (5–10 mTorr), the MFP of the sputtered particles was significantly shortened due to frequent collisions with residual gas molecules. These collisions dissipated the kinetic energy of the particles, causing them to reach the substrate with insufficient energy to overcome surface diffusion barriers. This limited atomic mobility on the substrate surface and hindered the formation of a well-ordered crystalline structure. Additionally, excessive collisions could result in increased scattering, causing non-uniform deposition and further reducing the overall crystallinity of the CrN buffer layer [37]. Thus, a process pressure of 5 mTorr was identified as optimal because it balanced sufficient energy for adatom surface diffusion at lower pressures and minimized energy dissipation due to frequent collisions at higher pressures, resulting in the highest CrN buffer layer crystallinity.

Figure 2d illustrates the crystallinity of the CrN buffer layers as a function of the RF power. The crystallinity improved as the power increased from 100 to 300 W, reaching its peak at 200 W with the lowest FWHM values for both the CrN buffer and GaN layer; however, it began to deteriorate at higher power levels. Increased power enhanced the electron density, resulting in more collisions on the target and substrate, which initially improved the film quality. Excessive energy at high power levels degraded the film quality, while insufficient energy at low power levels reduced the particle mobility on the substrate surface, resulting in poor crystallinity [38].

Under the optimal conditions (30% nitrogen gas ratio, 5 mTorr process pressure, 200 W RF power, and 400 °C deposition temperature), GaN exhibited the lowest FWHM values, which indicated the highest crystallographic quality. Notably, the FWHM values of GaN exhibited trends similar to those observed for the CrN crystallinity, indicating that the crystallographic quality of the CrN buffer layer directly impacted the GaN layer. These results emphasize the delicate balance required among the deposition parameters to achieve enhanced crystallinity, which is critical to improve the performance of GaN thin films in subsequent applications.

**Figure 3:**
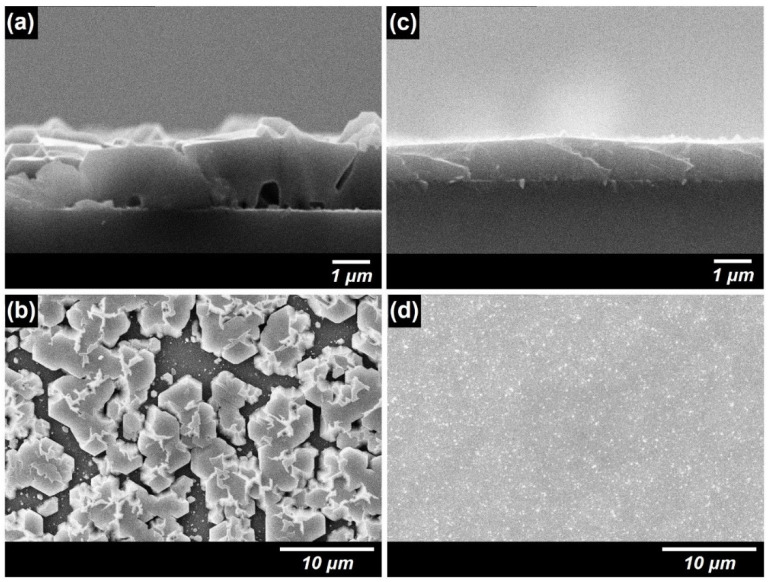
SEM images of the GaN layers on the CrN buffer layers: (**a**,**b**) without and (**c**,**d**) with the LT-GaN buffer layer; cross-sectional (**a**,**c**) and top view (**b**,**d**).

The deposition conditions for the growth of the GaN layer on the CrN buffer layer were determined through experiments using reactive RF magnetron sputtering. The morphology of GaN, which was grown on the CrN buffer layer using MOCVD, was observed using SEM, as shown in Figure 3a,b. For the sample without the LT-GaN nucleation layer, the cross-sectional SEM images revealed that GaN did not merge into a continuous film. Instead, large GaN islands were formed on the CrN buffer layer, leaving noticeable voids at the interface between the islands and the underlying buffer. This discontinuous growth behavior is attributed to insufficient nucleation sites on the CrN surface, which likely limited uniform adatom diffusion and promoted isolated island formation. The top-view SEM images further highlighted the non-uniform spatial distribution and irregular boundaries of these islands. Such three-dimensional nucleation behavior followed by film coalescence is consistent with the classical Volmer–Weber growth mechanism [39], which describes the preferential formation of 3D islands due to weak adatom–substrate interactions. This mechanism is frequently observed in GaN heteroepitaxy on lattice-mismatched substrates [40,41,42]. Inserting an LT-GaN nucleation layer between the CrN buffer layer and GaN significantly improved the film morphology, as shown in Figure 3c,d. The LT-GaN layer facilitated uniform nucleation across the surface, promoting continuous and void-free GaN film growth. This behavior reflects features of the Stranski–Krastanov growth mode, where a thin initial layer facilitates the transition into a three-dimensional growth regime [40]. The presence of this intermediate layer is thus essential in controlling the early-stage adatom behavior and surface coverage. The cross-sectional SEM analysis confirmed the formation of a uniform film with a thickness of approximately 1 μm and no detectable voids at the CrN/GaN interface. The top-view SEM image of this sample revealed a relatively smooth and uniform surface indicative of improved crystallographic ordering. Thus, the role of the LT-GaN layer was critical in this process. By serving as a nucleation layer, it enhanced the adhesion of GaN to the CrN buffer layer and facilitated the growth of a continuous GaN film by improving the initial interface for epitaxial alignment. These comparative results underscore the importance of an optimized nucleation layer in achieving high-quality GaN films.

**Figure 4:**
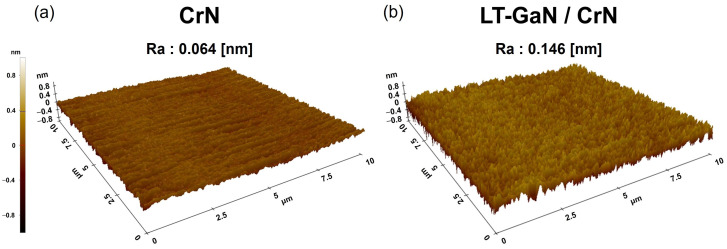
AFM 3D images and surface roughness comparison: (**a**) CrN buffer layer and (**b**) LT-GaN grown on the CrN buffer layer.

**Figure 5:**
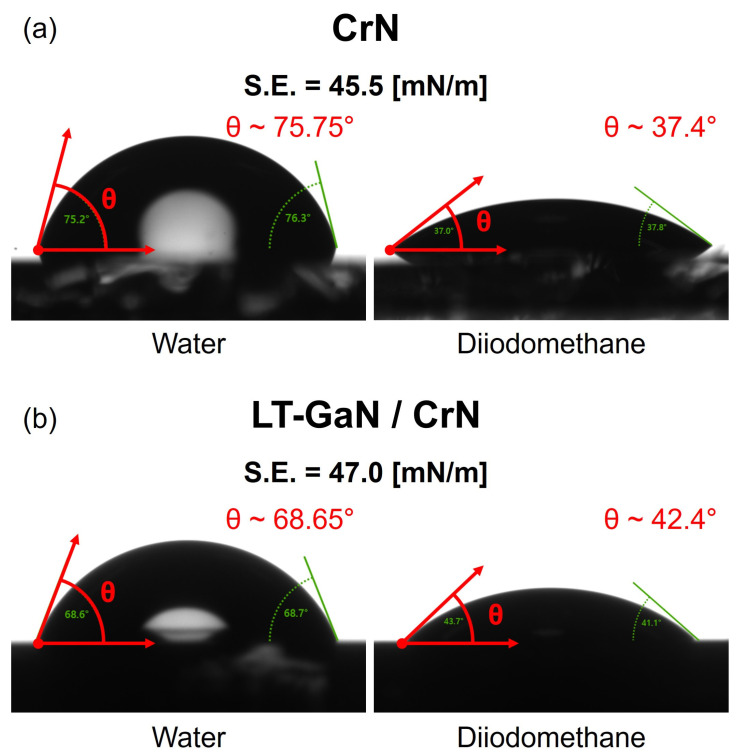
Contact angle measurements for surface energy comparison: (**a**) CrN buffer layer and (**b**) LT-GaN grown on the CrN buffer layer.

To thoroughly investigate their differences, surface morphology and roughness analyses of both the CrN buffer layer and the LT-GaN layer deposited onto it were conducted using AFM (Figure 4). Additionally, contact angle measurements were performed to analyze the surface energy variations (Figure 5). As shown in the AFM image in Figure 4a, the CrN buffer layer that was deposited onto the sapphire substrate exhibited a smooth surface with average roughness of 0.064 nm, indicating a uniform and well-ordered crystalline structure. However, after depositing the LT-GaN nucleation layer onto the CrN buffer layer, the surface morphology was transformed, displaying an increase in surface roughness to an average value of 0.146 nm. The AFM image shown in Figure 4b reveals the rough surface of the LT-GaN layer, which was characterized by small grains uniformly distributed across the substrate. This morphological change suggested that the LT-GaN layer effectively modified the surface conditions of the CrN buffer, providing enhanced nucleation sites that facilitated the subsequent epitaxial growth of GaN. The surface energy analysis using AFM was used to further explain the differences in surface roughness.

The contact angle measurements indicated that the CrN buffer layer had surface energy of 45.5 mN/m, while the LT-GaN/CrN buffer layer had slightly higher surface energy of 47.0 mN/m (Figure 5). In addition, when an LT-GaN nucleation layer was introduced on top of the CrN buffer, the contact angle of water decreased from 75.75° to 68.65°, and that of diiodomethane increased from 37.4° to 42.4°. Changes were observed for both liquids, indicating a qualitative shift in the polar and dispersive components of the surface energy. The increase in surface energy correlated with improved wetting properties, because the higher surface energy promoted particle spreading on the substrate surface. This behavior can be explained using the Owens–Wendt–Rabel–Kaelble method, which enables the calculation of the surface free energy by resolving it into its polar and dispersive components based on contact angle measurements. Based on the contact angle data, Young’s equation (Equation (1)) and the Owens–Wendt equations (Equations (2) and (3)) were used to calculate the surface free energy:

(1) $$\gamma_{SG}=\gamma_{SL}+\gamma_{LG}\cos\theta$$

(2) $$\gamma_{SG}=\gamma_{SL}+\gamma_{LG}-2\sqrt{\gamma_{SG}^{d}\cdot \gamma_{LG}^{d}}-2\sqrt{\gamma_{SG}^{p}\cdot \gamma_{LG}^{p}}$$

(3) $${\gamma_{SG}}_{total}={\gamma_{SG}}^{d}+{\gamma_{SG}}^{p}$$

where $\gamma_{SL}$ represents the solid–liquid interfacial tension, $\gamma_{SG}$ denotes the solid–gas surface energy, and $\gamma_{LG}$ corresponds to the liquid–gas interfacial energy. The superscript ‘*p*’ indicates the polar component, while ‘*d*’ represents the dispersive component of the surface energy [43,44]. Although the surface energy difference between LT-GaN/CrN and CrN is relatively modest, even such a minor variation can meaningfully influence the early stages of film growth. According to classical nucleation theory, higher substrate surface energy improves wetting by reducing the contact angle of the nucleating species, which in turn facilitates the thermodynamic conditions necessary for stable nucleus formation. This enhancement in interfacial compatibility can promote the more uniform spatial distribution of nucleation sites, ultimately leading to improved film continuity. In agreement with this interpretation, the SEM images (Figure 3) reveal that GaN layers grown on LT-GaN/CrN exhibit continuous and coalesced morphologies, while those deposited directly on CrN display fragmented, island-like growth indicative of limited nucleation coverage.

The role of the LT-GaN nucleation layer was pivotal in optimizing the growth environment for GaN. By increasing the surface roughness and energy, the LT-GaN layer created favorable conditions for epitaxial growth, functioning as a bridge to overcome the lattice mismatch between GaN and the CrN buffer layer. For example, the rougher surface morphology provided more active sites for nucleation, while the higher surface energy supported particle diffusion, enabling GaN to laterally spread across the surface. This lateral growth mechanism was essential to reduce the dislocation density, which significantly impacted the overall crystallographic quality of the GaN film. Moreover, the improved wetting and nucleation properties associated with the LT-GaN layer mitigated the formation of voids at the interface between the CrN buffer layer and GaN film. This phenomenon was corroborated in the SEM analysis, where the GaN film grown on the LT-GaN nucleation layer merged into a continuous and void-free film, unlike the island-like morphology observed for GaN grown directly on the CrN buffer layer. The enhanced growth dynamics provided by the LT-GaN layer contributed to a higher-quality GaN film with fewer structural defects and improved optical and electronic properties [4,45]. These findings highlight the critical role of the LT-GaN layer not only in nucleation but also in promoting ideal epitaxial growth conditions, rendering it an indispensable component in achieving high-quality GaN films for advanced applications.

**Figure 6:**
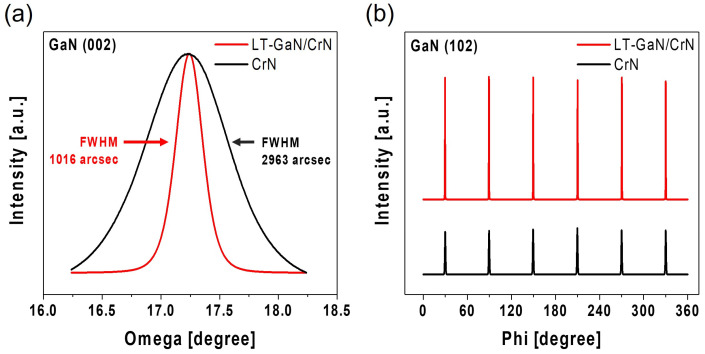
HR-XRD results of GaN grown on CrN and LT-GaN/CrN: (**a**) ω-rocking curves of GaN (002); (**b**) φ-scans of GaN (102).

The out-of-plane crystalline quality of the GaN films was also evaluated using ω-rocking curve measurements of the GaN (002) reflection (Figure 6a). The GaN film grown directly on CrN buffer layer exhibited the FWHM value of 2963 arcsec, indicating a relatively broad distribution of crystallographic tilt. In contrast, the introduction of an LT-GaN nucleation layer reduced the FWHM significantly to 1016 arcsec, suggesting enhanced vertical alignment and reduced mosaicity in the GaN crystal structure. This result corroborates the improvements observed in the film morphology and lateral crystallinity and confirms the beneficial role of the LT-GaN layer in promoting higher-quality epitaxial growth. Additionally, X-ray phi scans of the GaN (102) reflection were employed to assess the symmetry of the crystals (Figure 6b). Although morphological differences in GaN grown on different buffer layers were observed (Figure 3), the X-ray phi scan results revealed that the GaN in both samples exhibited six-fold symmetry. Thus, the GaN layers maintained consistent crystallographic alignment regardless of the buffer layer used, reflecting stable epitaxial growth conditions. Notably, the GaN layer grown on the LT-GaN/CrN buffer layer exhibited a higher intensity in the X-ray phi scan compared to the GaN layer grown directly on the CrN buffer layer. This observation suggests improved crystallinity and a more uniform lattice structure in the presence of the LT-GaN nucleation layer. The LT-GaN layer facilitated a reduction in lattice mismatch and improved the interface quality, promoting the enhanced alignment of the GaN lattice.

**Figure 7:**
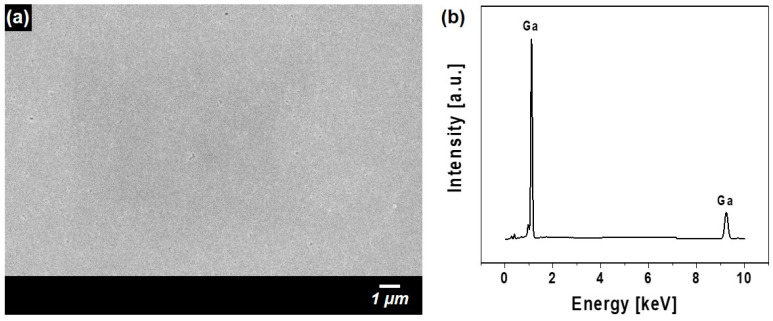
(**a**) SEM image of the backside of the GaN film separated via the CLO process. (**b**) EDX analysis of the separated GaN film.

The backside of the exfoliated GaN film was observed using SEM, which revealed a characteristic void structure (Figure 7a). The EDX results shown in Figure 7b further confirm the absence of Cr signals. Because only Ga was detected, the CrN buffer layer was probably completely removed during the CLO process. Although the voids observed on the GaN backside may be related to the removal of the CrN buffer layer, their exact origin remains unclear and requires further investigation. However, these results demonstrate that the CLO process, which used CrN as the sacrificial layer, could effectively separate the GaN film from the sapphire substrate.

## 4. Conclusions

The crystallographic and morphological effects of varying deposition conditions for CrN buffer layers were analyzed, focusing on their impacts on GaN thin film epitaxy. Using detailed parameter optimization, a substrate temperature of 400 °C, nitrogen gas ratio of 30%, process pressure of 5 mTorr, and RF power of 200 W yielded CrN buffer layers with superior crystallinity, thereby serving as a robust foundation for high-quality GaN growth. The introduction of an LT-GaN nucleation layer directly on CrN further improved the GaN film quality and facilitated a smoother transition to subsequent HT-GaN growth, underscoring its critical role in the epitaxial process. These findings highlight the interplay between buffer layer engineering and nucleation layer introduction in achieving optimal GaN thin films. While this study primarily focused on crystallographic and morphological trends to evaluate the suitability of the CrN and LT-GaN layers for GaN epitaxy, further investigation is required to quantify the dislocation densities and assess the implications for device-level performance. Additionally, surface chemical analysis, including the evaluation of oxygen incorporation at the GaN/CrN interface, will be conducted in future work to clarify the interfacial stability. These directions will support the continued development of lift-off-capable GaN device structures and high-quality heteroepitaxy on engineered buffer layers.

## Figures and Tables

**Table 1:** Temperature-dependent lattice mismatch of CrN thin films with sapphire and GaN.

Sample Code	2θ (°)	d (Å)	a_0_ (Å)	Lattice Mismatch	
				with Al_2_O_3_ (0001) (%)	with GaN (002) (%)
CrN 300 °C	37.402	2.402	4.161	7.11	−7.73
CrN 400 °C	37.350	2.406	4.167	7.26	−7.61
CrN 500 °C	37.104	2.421	4.193	7.94	−7.02
CrN 600 °C	37.071	2.423	4.197	8.03	−6.94

## Data Availability

The original contributions presented in this study are included in the article. Further inquiries can be directed to the corresponding author.

## Abbreviations

The following abbreviations are used in this manuscript:

GaN	gallium nitride
LED	light-emitting diode
MOCVD	metal–organic chemical vapor deposition
LT	low temperature
LLO	laser lift-off
CLO	chemical lift-off
RF	radio frequency
HT	high temperature
XRD	X-ray diffraction
EDX	energy-dispersive X-ray spectroscopy
FWHM	full-width at half maximum
AFM	atomic force microscopy
SEM	scanning electron microscopy
MFP	mean free path
